# Performance of routine surveillance diagnostics of external ventricular drain-associated infections in a critical care setting: a retrospective cohort study

**DOI:** 10.1186/s12879-025-11006-1

**Published:** 2025-05-02

**Authors:** Marcus Ståhlberg, Jonas Blixt, Christer Mehle, Viveca Hambäck Hellkvist, Christian G. Giske, Eddie Weitzberg, David W. Nelson

**Affiliations:** 1 Function Perioperative Medicine and Intensive Care, Karolinska University Hospital, Stockholm, Sweden; 2 Department of Physiology and Pharmacology, Karolinska Institutet, Stockholm, Sweden; 3 Faculty of Medicine, Department of Clinical Microbiology, Infectious Diseases, Umeå University, Umeå, Sweden; 4 Division of Clinical Microbiology, Department of Laboratory Medicine, Karolinska Institutet, Stockholm, Sweden; 5 Department of Clinical Microbiology, Karolinska University Hospital, Stockholm, Sweden

**Keywords:** External ventricular drain associated infections, Ventriculostomy associated infections, External ventricular drain associated infection surveillance

## Abstract

**Introduction:**

External ventricular drains (EVDs) are crucial for treating neurocritically ill patients but are complicated by feared EVD-associated infections (EVDIs) in up to 35% of all inserted drains, contributing significantly to morbidity, mortality and account for a significant proportion of intensive care unit (ICU) antibiotic use. However, the lack of a universal definition for EVDIs leads to inconsistent diagnostic criteria across studies, with a concern of substantial overtreatment with broad-spectrum antibiotics. This study aimed to evaluate if current EVDI surveillance parameters can be optimized to better distinguish true from suspected EVDI.

**Methods:**

We conducted a retrospective cohort study at the Karolinska University Hospital ICU, including all patients treated with EVDs between 2006 and 2023, excluding patients with primary central nervous system (CNS) infections. EVDI surveillance included biweekly sampling and cultures from cerebrospinal fluid (CSF). Patients were categorized as no infection (NI), suspected infection (SI), or verified infection (VI) based on culture results and treatment status. We employed classification and regression analyses to identify predictors of VI.

**Results:**

Among 1,828 patients with EVDs, 29.8% were initiated on antibiotic treatment due to suspected infection and 4.1% were found to have culture confirmed infections. The main finding is that current accepted diagnostic parameters cannot distinguish aseptic inflammation from true EVDI. In multivariable logistic analysis the best models exhibited low accuracy, with a pseudo-$R^2$ of only 0.06. CSF lactate was the most important metric in a univariable setting, however with a cut-off of 8.9 mmol/L it showed low discrimintive ability and limited clinical utility.

**Conclusions:**

In this study we evaluate current accepted EVDI surveillance methods in, to our knowledge, the largest cohort of paired samples to date. We find that current surveillance parameters cannot distinguish aseptic CNS inflammation from true EVDIs in an ICU setting. This contributes to a significant antibiotic overtreatment, with 25% of our entire cohort being unnecessarily initiated on broad-spectrum antibiotics, a number we expect can be generalized. We identify a large clinical problem with consequences on both a individual and population level, and recommend that future research focus on evaluating new techniques, such as fast bedside sequencing methods.

**Supplementary Information:**

The online version contains supplementary material available at 10.1186/s12879-025-11006-1.

## Introduction

External ventricular drains (EVD) are commonly used in the treatment of neurocritically ill. EVD use is complicated by high rates of infection (EVDI) which have been associated with increased mortality and considerable long-term neurological deficits, as well as increased hospital stay and costs [1–5]. Current EVDI surveillance methods are based on indirect clinical and biochemical signs of infection, but these are ambiguous in the critically ill [6–10].

Currently, there is no single established definition of EVDI, and various diagnostic criteria are used across studies. Thus, inference of published results is difficult, resulting in a lack of generalizability [11, 12]. Inconsistencies in diagnostic indicators, such as pleocytosis and lactate, contribute to the challenges of forming a consensus on the appropriate management of EVDI [10, 13, 14]. In addition, EVDIs are likely to be heterogeneous infections affected by patient characteristics, hygienic routines, care settings, and microbiological profiles, which are all site dependent factors likely to affect both clinical and biochemical presentation in the critically ill [14, 15]. As a result, the currently most influential guidelines issued by the Infectious Diseases Society of America (IDSA) in 2017 are based on contradictory findings, and recommendations are generally supported by weak evidence [6]. To date, no single indirect diagnostic parameter has been identified to, by itself or in the aggregate, reliably identify or predict EVDIs [6, 16–19].

Thus, the tools available to the clinician to guide EVDI targeted therapy are limited and lack specificity in an intensive care unit (ICU) setting. Bacterial culture is the gold standard for establishing a diagnosis, but is not readily available to guide treatment, with specifically negative cultures taking 12 days to finalize at our institution [6, 9, 20, 21]. Although infections are unlikely to be missed with rigorous surveillance, EVDI targeted therapy is often initiated empirically, with considerable overtreatment with broad-spectrum antibiotics as a result [8, 11, 22–25]. There is a pressing need to reduce unnecessary treatment with broad-spectrum antibiotics both from a patient point of view and to counteract the development of antimicrobial resistance.

In this extensive retrospective cohort study, we evaluated the performance of common indirect EVDI surveillance metrics over a 17-year period, related to the ability to differentiate between clinically suspected EVDIs and culture-confirmed EVDIs in an ICU setting.

## Methods

### Setting and participants

The study included all adult patients (18 years or older) treated with EVDs in the ICU of Karolinska University Hospital in Solna between 2006 and 2023. Karolinska serves as the only trauma and neurosurgical referral center for approximately 2.5 million inhabitants in the greater Stockholm region. Infection prevention measures maintained consistently throughout the study included EVD insertion under sterile conditions in an operating theater with a single dose prophylactic antibiotics, daily dressing changes, daily cleaning of the insertion site with chlorhexidine, and aseptic cerebrospinal fluid (CSF) sampling performed by trained ICU nurses. Neither silver nor antibiotic-impregnated catheters were routinely used during the study period. Patients with central nervous system (CNS) infections as primary admission diagnoses were excluded. The study was approved by the Swedish Ethical Review Authority (DNR 2017/1544 - 31/1, DNR 2018/1701 - 32, DNR 2024–07464- 02).

### Data collection, structure, and missing data

All data was retrieved from electronic patient journal database systems. Clinical data included results from CSF-, blood-, and microbiology analyses as well as antibiotic therapy, temperature, and reported Glasgow Coma Scale (GCS). Blood was drawn daily and CSF was sampled bi-weekly for routine EVDI surveillance or as part of patient work up. Demographic data included age, patient sex, admission diagnosis. Data on administered antibiotics, ICU length of stay, length of catheterization, and mortality were also retrieved.

The groups were imbalanced due to the relatively low incidence rate of culture confirmed EVDIs. Final analyses were performed on the unbalanced dataset, with sensitivity analyses employing both under- and oversampling techniques yielding similar results. Multiple regression imputation (‘mice’ v 3.14.0) was used to handle missing data for the regression analyses. The glucose ratio was not routinely analyzed and estimation using linear interpolation was used when a matching blood glucose value within 30 minutes of a CSF sample was missing as the sampling frequency was judged high enough for such an approximation. Analyses of procalcitonin (PCT), neuron-specific enolase (NSE), and S100B were dependent on specific patient diagnoses, resulting in a high degree of missing data in our cohort. GCS was calculated at least three times per day unless the patient was under continuous sedation. Temperature readings were either continuous via urinary catheter (central) or manually recorded using tympanic temperature (peripheral), and are not distinguished in our dataset.

### EVDI classification

During the study period, routine EVDI surveillance consistently involved a comprehensive evaluation of clinical presentation alongside biochemical CSF parameters obtained from bi-weekly sampling or more often when clinically indicated. The CSF parameters analyzed included cell counts, protein levels, lactate concentration, glucose levels, and bacterial cultures. Treatment protocols adhered to local and national guidelines aligned with the latest IDSA recommendations, taking into consideration clinical presentations and temporal patterns in key CSF metrics, particularly glucose, lactate, and granulocyte counts [6]. The decision to initiate EVDI targeted therapy rested with the treating clinician, supported by infectious disease consults. In summary, EVDI targeted therapy in this study is a proxy for strong clinical suspicion based on the aggregate evaluation by the intensivist and infectious disease consult of risk factors, epidemiology, clinical, and biochemical factors.

Patients were retrospectively classified into three groups based on microbiology results and EVDI targeted therapy status:

**Verified infection (VI):**

1. One or more positive CSF bacterial culture *and*
2. EVDI targeted antibiotic therapy

**Suspected infection (SI):**

1. Negative CSF bacterial culture *or* contamination *and*
2. EVDI targeted antibiotic therapy

**No infection (NI):**

1. Negative CSF bacterial culture *or* contamination *and*
2. No EVDI targeted antibiotic therapy

Classification criteria were established prior to the initiation of the study. A manual review was conducted for all patients with positive cultures. Consistent with the IDSA EVDI guidelines, contamination was defined as a positive CSF bacterial culture in a patient who was never started on EVDI targeted therapy (i.e lacking clinical or biochemical signs indicative of infection), or where there was a clear temporal disconnect between the positive culture and EVDI targeted antibiotic therapy. The day on which SI and VI patients commenced EVDI targeted antibiotic therapy is henceforth referred to as the index date. For NI patients, the index date corresponded to the date of the sample with the highest granulocyte count.

### Derived variables

The CSF-to-blood glucose ratio (glucose ratio) was calculated for all CSF samples with corresponding blood glucose measurements obtained within 30 minutes. Delta 1D and Delta 2D were utilized to represent temporal trends, and exponential approximation was employed to address missing data points through interpolation.

**GE ratio:** The ratio of granulocytes to erythrocytes.

**GM ratio:** The ratio of granulocytes to monocytes.

**Glucose ratio:** The ratio of CSF glucose to glucose in blood.

**Delta:** Changes within the same day as the index date. Used for clinical parameters to identify clinical deterioration.

**Delta 1D:** Changes compared to the day immediately preceding the index date.

**Delta 2D:** Changes compared to two days before the index date.

### Statistical analyses

Categorical variables are presented as counts and percentages, and continuous variables as means and standard deviation (SD) or medians and interquartile range (IQR). Infection rates were normalized to infections per 1000 catheter days. Normality was assessed using visual inspection. Log- or square root transformation to approach a normal distribution was used when necessary. Descriptive analyses were performed on unimputed data with the Chi-square test and the non-parametric Wilcoxon rank sum (WRS) test to evaluate categorical variables and continuous variables, respectively. The non-parametric WRS test was utilized to evaluate differences between groups.

Univariate logistic regression analyses were utilized to evaluate variables potential to differentiate between SI and VI. Multivariate logistic regression analyses were utilized to evaluate the potential of a predictive model for VI and SI. Linear regression analyses were utilized to evaluate to which degree granulocytes and lactate can be explained by other CSF variables. Conditional density (CD) plots were used to visually inspect data and to evaluate changes in the distribution of the three groups across the range of chosen variables. One sample per patient (which best correlated in time with the index date) was chosen for regression analyses.

Final analyses were performed on the unbalanced dataset, with sensitivity analyses employing both under- and oversampling techniques yielding similar results. Multiple regression imputation (‘mice’ v 3.14.0) was used to handle missing data for the regression analyses. Missing data are presented in Table 3 and are generally low, with a few exceptions. The glucose ratio was not routinely analyzed and estimation using linear interpolation was used when a matching blood glucose value within 30 minutes of a CSF sample was missing.

Regression analyses focused on three objectives, first to identify significant predictors to differentiate between EVDI groups, second to identify significant predictors of CSF granulocytes, and third to identify significant predictors of CSF lactate. Covariates exhibiting significance levels of $p < 0.2$ in a univariable regression setting were carried forward to multivariable regression analyses. Variable selection was performed using backwards stepwise selection based on significance levels. The variable selection was further corroborated via Aikaike information criterion (AIC) step wise regression. Covariates with a high proportion of missing data ($> 30\%$) were not included in the multivariable regression analyses. Univariable and multivariable logistic regression model performances were evaluated using bias-adjusted pseudo $R^2$ (Nagelkerke). Classification analysis was performed using regression trees (‘rpart’ v 4.1.15).

All statistical analyses were performed using R version 4.3.3 (2024 - 02- 29).

## Results

Between 2006 and 2023, 1830 patients were treated with EVDs for non-infectious etiologies. Demographics are presented in Table 1. The most common admission diagnosis was subarachnoid hemorrhage (SAH). Two patients with primary CNS infection were included after manual inspection of all patients with positive cultures. The result was 75 patients with culture-confirmed EVDI (4.1%, VI), 470 with suspected infection (25.7%, SI), and 1280 with no infection (70%, NI), yielding 3.72 VI and 23.1 SI per 1000 catheter days. A total of 8072 paired CSF samples and cultures were collected, with 3579 from SI or VI patients. Age and gender distributions were similar across groups, while total catheterization days and ICU lengths of stay were higher in SI and VI groups compared to NI. Mortality rates showed no differences between groups (Table 1, Chi-squared $>.6$).

**Table 1:** Cohort demographics

	NI	SI	VI
**Demographics**			
Total	1283	470	75
Female gender (%)	51	47	54
Age (mean)	55.4	55.6	53
ICU LoS (days, mean)	12.0	17.7	19.0
EVD duration at index date (days, mean)	NA	6.3	6.9
EVD duration total (days, mean)	10.0	14.0	16.2
EVDI treatments per 1000 catheter days	NA	23.1	3.7
Antibiotics prior to index date (%)	NA	57	47
90 day mortality (%)	14.1	15.9	10.5
180 day mortality (%)	16.7	17.8	14.5
**Admission diagnoses**			
SAH	530	199	33
ICH	241	74	6
TBI	146	82	5
Other Cerebral Diseases	272	89	22
Other Non-Cerebral Diseases	90	27	10

ICU LoS is intensive care unit length of stay. EVD is external ventricular drain. NI, SI and VI is no infection, suspected infection, and verified infection, respectively. SAH is subarachnoid hemorrhage. ICH is intracerebral hemorrhage. TBI is traumatic brain injury. NA is not applicable. Index date is the day EVDI targeted antibiotic therapy was initiated

Patients with EVDs made up approximately 10% of the total ICU population in our institution during the study period. Among these, the 30% of all EVD patients that received EVDI targeted therapy accounted for 15% of the total antibiotic usage in all ICU patients, measured in terms of days of antibiotic treatment. Antibiotic therapy was initiated, on average, at day six post-catheterization for SI and VI patients, with no significant differences in prevalence of non-EVDI targeted antibiotic therapies prior to CSF sampling ($p> .05$).

Bacteria grew in 241 CSF bacterial cultures, with 166 evaluated as clinically relevant. 75 positive cultures were classified as contamination (known contaminants, not treated with EVDI targeted antibiotics). Five patients with contaminated cultures had multiple positives but did not receive EVDI treatment, showing no signs of infection, with cultures becoming positive after 8–12 days. These patients are believed to be correctly classified as NI with repeated cultures suggesting distal port contamination, rather than EVDI. Antibiotic resistance was rare, with only 12 cases of ESBL-producing Klebsiella pneumoniae identified and no MRSA was identified. The most common pathogen was coagulase-negative staphylococci, followed by Cutibacterium acnes and Klebsiella pneumoniae (Table 2). EVDI-specific antibiotic treatment accounted for 15% of the total antibiotic days for all ICU-admitted patients since 2006.

**Table 2:** Culture microbiology results

Culture microbiology	Clinically relevant	Contamination	Total
**Gram positive**			
Coagulase negative staphylococci	76	34	110
Cutibacterium acnes	24	30	54
Bacillus spp.	6	2	8
Staphylococcus aureus	5	2	7
Micrococcus	3	1	4
Other	9	4	12
**Gram negative**			
Klebsiella Pneumoniae	21	0	21
Stenotrophomonas maltophilia	12	1	13
Serratia marcescens	6	0	6
Acinetobacter spp.	1	0	1
Other	2	1	3
Total	165	75	240

Classification as clinically relevant or contamination was done in accordance to our predefined definition for contamination and is based on EVDI treatment status. A positive culture in a patient not initiated on EVDI targeted antibiotic treatment, or in case of a clear temporal disconnect between a positive culture and treatment, was classified as a contaminant. In our study, EVDI treatment status is a proxy for strong clinical suspicion, based on the constitute judgment of the intensivist and infectious disease consult in charge each individual case, following the evaluation of risk factors, epidemiology, clinical, and biochemical signs of infection

**Fig. 1:**
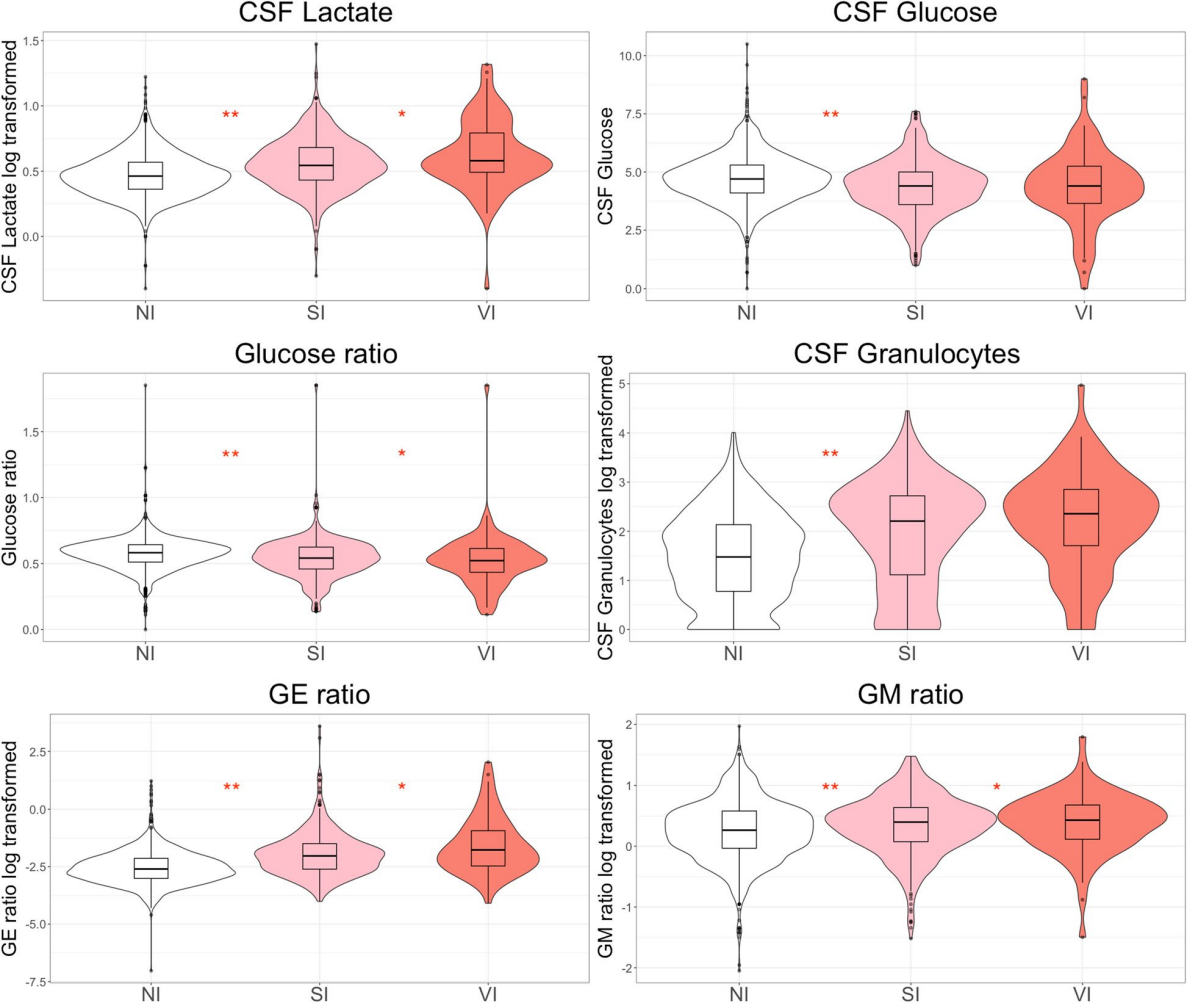
Violin plots visualizing the distribution of data for six parameters of interest for patients classified as SI and VI. Box plot overlays to improve visualization of the means and outliers. Some variables were log transformed due to outliers to improve visualization where indicated. The asterisks show significance levels for the Wilcoxon rank sum test between NI vs SI and VI (NI vs AB in Table 3), and SI vs VI, respectively. * indicate $p < 0.05$. ** indicate $p < 0.01$. CSF is cerebrospinal fluid. The glucose ratio is the ratio of CSF glucose to blood glucose. The GE ratio is the ratio of granulocytes to erythrocytes in CSF. The GM ratio is the ratio of granulocytes to monocytes in CSF

**Fig. 2:**
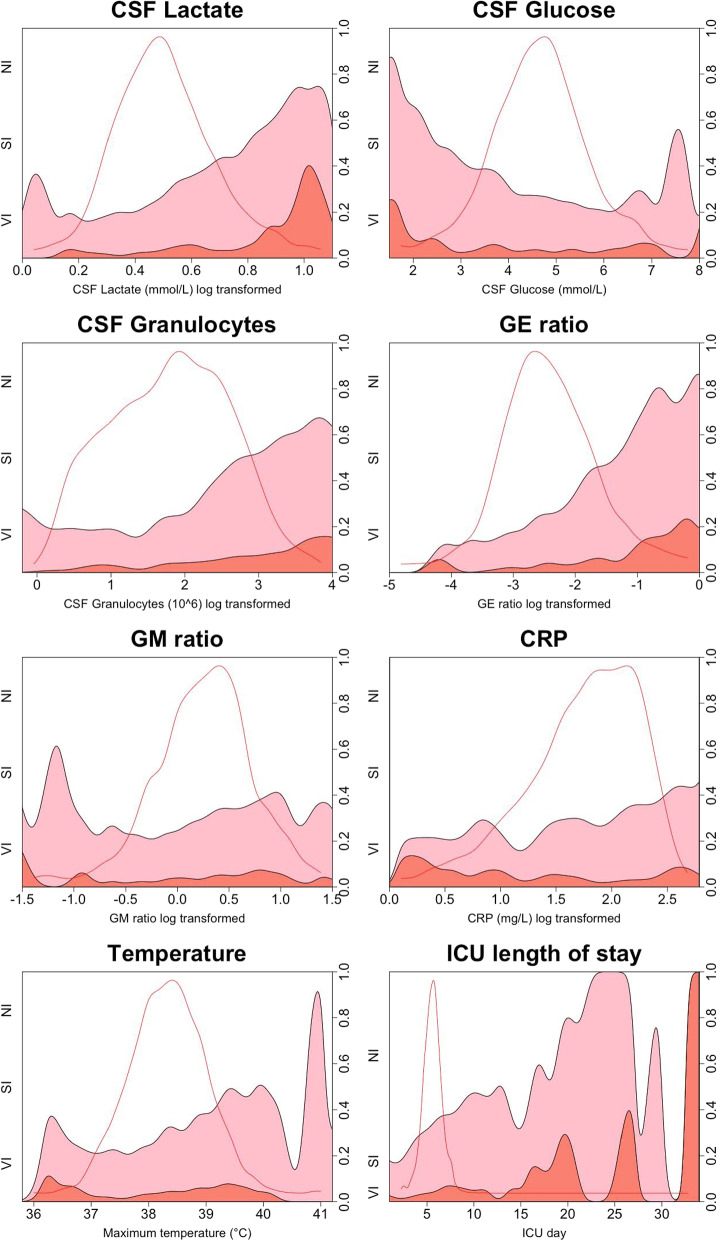
Conditional density plots visualizing chosen biochemical and clinical variables and their relations to no EVDI, suspected EVDI, and culture confirmed EVDI at levels along the x axis. NI, SI, and VI, are represented as white, pink, and salmon, respectively. Density plot overlays (red line) showing the data distribution of the underlying data with an arbitrary y axis, but where area will sum to 1. CSF is cerebrospinal fluid. The GE ratio is the ratio of granulocytes to erythrocytes in CSF. The GM ratio is the ratio of granulocytes to monocytes in CSF. CRP is C-reactive protein in blood

Central tendencies and the results of comparative analyses, univariable logistic regression analyses, and linear regression analyses at the index date, defined as the day EVDI targeted therapy was initiated, are presented in Table 3 (corresponding results for log-transformed variables are presented in supplementary table 1). *P*-values are presented for comparative analyses while pseudo-$R^2$ is used for regression analyses with corresponding *p*-values being presented in text. Selected variable distributions are visualized with box and violin plots in Fig. 1, and with CD plots in Fig. 2. Missing data are presented in Table 3 and are generally low, with a few exceptions.

**Table 3:** Comparisons of central tendency (CT) between groups and results of univariable logistic and linear regression analyses

	Central tendency				Wilcoxon Rank Sum *(p)*		Logistic Regression *(R*^*2*^*)*		Linear Regression *(R*^*2*^*)*	
	NI	SI	VI	Missing	NI vs AB	SI vs VI	NI vs AB	SI vs VI	CSF Granulocytes	CSF Lactate
CSF Granulocytes ($10^6$/L)	29 (5–135)	160 (12–522)	226 (50–713)	0	**.0000**	.0767	**.0518**	.0134	*NA*	**.0694**
CSF Monocytes ($10^6$/L)	22 (4–85)	60 (13–185)	82 (28–229)	5	**.0000**	.1657	**.0366**	.0178	**.5418**	**.0827**
CSF Erytrocytes ($10^6$/L)	13750 (2400–52725)	14000 (2076–49300)	13800 (1350–40100)	0	.4724	.5340	.0032	.0049	.0000	.0014
CSF Lactate (*mmol/L*)	3.2 (1.4)	4.1 (2.4)	5.2 (3.7)	6	**.0000**	.0243	**.0823**	**.0296**	**.0694**	*NA*
CSF Glucose (*mmol/L*)	4.8 (1.0)	4.3 (1.2)	4.3 (1.6)	10	**.0000**	.8820	**.0378**	.0005	**.0055**	**.0797**
CSF Albumin (*mg/L*)	238 (113–496)	360 (191–618)	342 (145–718)	7	**.0000**	.6623	.0056	.0024	**.0359**	**.2474**
GE ratio	1.9 (.6–6.2)	7.0 (2.0–26.0)	13.3 (3.0–97.0)	0	**.0000**	.0119	.0219	.0009	**.0086**	**.0291**
Glucose ratio	.58 (.11)	.53 (.15)	.50 (.14)	22	**.0000**	.0291	**.0400**	.0147	**.0069**	**.1630**
GM ratio	1.7 (.8 - 3.1)	2.1 (.9 - 3.9)	2.7 (1.3–4.8)	14	**.0001**	.0423	.0038	.0090	**.0371**	**.0307**
B Glucose (*mmol/L*)	8.4 (1.8)	8.3 (1.8)	8.8 (2.4)	13	.9250	.4692	.0001	.0138	.0001	**.0152**
B Leukocytes ($10^9$/L)	10.9 (8.7–13.6)	11.7 (9.0–14.5)	11.4 (9.3–14.7)	5	**.0041**	.7870	.0062	.0091	.0003	**.0217**
B CRP (*mg/L*)	67 (26–136)	103 (42–184)	53 (26–181)	5	**.0000**	.0242	**.0253**	.0022	.0003	.0002
B PCT (µg/L)	.15 (.08-.40)	.21 (.10-.50)	.14 (.10-.40)	58	.0461	.6431	.0010	.0020	.0001	.0025
B NSE (µg/L)	14 (11–20)	15 (11–20)	17 (12–23)	58	.7755	.2514	.0005	.0018	.0001	.0010
B s100b (µg/L)	.10 (.06-.20)	.08 (.05-.15)	.11 (.06-.16)	22	**.0009**	.2701	.0002	.0037	.0012	**.0137**
GCS delta	.17 (2.12)	-.04 (2.25)	-.40 (2.24)	34	.1467	.1217	.0022	.0040	.0006	.0007
Motor GCS delta	.08 (.94)	-.01 (1.10)	-.04 (1.07)	34	.1467	.2871	.0012	.0020	.0003	.0004
Temperature max (°C)	38.1 (.8)	38.4 (.8)	38.4 (.9)	12	**.0000**	.9111	**.0292**	.0008	.0000	.0021
Temperature delta (°C)	.14 (1.55)	-.07 (1.80)	.01 (1.72)	12	.0353	.7909	.0035	.0007	.0019	.0031
ICU LoS at index date (days)	3 (2–6)	5 (3–9)	6 (3–9)	0	**.0000**	.6044	**.0754**	.0055	**.0043**	.0002
ICU LoS total (days)	10 (5–17)	17 (11–23)	18 (13–26)	0	**.0000**	.1349	**.1135**	.0043	.0035	.0034
EVD duration at index date (days)	3 (1–6)	5 (2–8)	6 (3–9)	0	**.0000**	.2557	**.0319**	.0003	.0006	.0000
EVD duration total (days)	8 (4–14)	13 (8–18)	15 (10–21)	0	**.0000**	.0114	**.0726**	.0028	.0027	.0034
Lactate delta 1D (*mmol/L*)	.04 (.32)	.24 (1.03)	.46 (1.76)	6	**.0000**	.3382	**.0325**	.0069	**.0352**	**.1904**
Lactate delta 2D (*mmol/L*)	.04 (.43)	.34 (1.44)	.77 (2.27)	6	**.0000**	.2641	**.0433**	.0126	**.0428**	**.2114**
Granulocyte delta 1D ($10^6$/L)	0 (0–11)	0 (0–147)	1 (0–285)	0	.0491	.4204	**.0234**	.0099	**.8145**	**.0373**
Granulocyte delta 2D ($10^6$/L)	0 (0–17)	0 (0–210)	26 (0–369)	1	**.0007**	.4280	**.0291**	.0101	**.8293**	**.0315**
Prior antibiotics	*NA*	*NA*	*NA*	*NA*	*NA*	*NA*	**.0680**	.0080	.0019	.0029

CT are presented as mean (SD), or as median (IQR). Missing is number of data points missing before imputations for SI and VI patients in percent. The Wilcoxon rank sum test is presented as significance levels (p). The results from logistic and linear regression are presented as bias adjusted pseduo-$R^2$ (Nagelkerke). Results in bold denotes a statistical significance level of $p < 0.01$. NI, SI, and VI are no infection, suspected infection, and verified infection, respectively. AB are those treated with antibiotics for EVDI (SI and VI merged). CSF is cerebrospinal fluid. Prefix B indicates results related to blood analyses. CSF granulocytes and CSF lactate columns are the results from linear regression analyses with CSF granulocytes and CSF lactate as dependent variables. GM ratio is the ratio of granulocytes to monocytes in CSF. GE ratio is the ratio of granulocytes to erythrocytes in CSF. CRP is C-reactive protein. PCT is procalcitonin. NSE is neuron-specific enolase. GCS is Glascow Coma Scale. ICU LoS is the ICU length of stay. EVD is external ventricular drain. The delta represent changes within the same day as the index date for clinical parameters. The delta 1D and 2D are changes from one and two days prior to the index date

### Differences between EVDI targeted therapy recipients and non-recipients

Naturally, all CSF parameters, except erythrocytes, were significantly lower in patients who did not receive EVDI targeted therapy (NI) compared to those who did (SI and VI, AB). No significant differences were observed in actual GCS scores or changes in GCS between these groups. These findings align with the expectation that the initiation of EVDI targeted therapy is typically guided by clinical signs and abnormal CSF findings at our institution.

### CSF Lactate

CSF lactate levels were significantly higher in VI patients compared to SI patients (5.2 vs 4.1, $p=0.02$, Table 3). CSF lactate was a significant predictor of VI in a univariable logistic analysis ($p < 0.01$, Table 3), however, yielded a low pseudo-$R^2$ of 0.03, indicating limited diagnostic utility for CSF lactate as a marker of culture confirmed EVDI. This is further illustrated in Fig. 2, where the proportion of VI increases arguably more than SI with higher CSF lactate levels. The 1D and 2D CSF lactate delta were not significant predictors of VI in a univariable logistic analysis ($p=0.15$ and $p=0.06$, respectively), reinforcing that CSF lactate is not a reliable marker for EVDI surveillance.

A linear regression model fitted to the full cohort, predicting CSF lactate levels using granulocytes, monocytes, erythrocytes, glucose, and albumin as predictors, yielded a pseudo-$R^2$ of.39, thus explaining 39% of the variance in CSF lactate. A model based on only SI and VI patients yielded a pseudo-$R^2$ of.5. This indicates that lactate levels are highly related to other CSF variables, which by themselves do not reliably differentiate between SI and VI.

### CSF Glucose and the glucose ratio

No significant differences in CSF glucose levels between SI and VI patients were observed ($p=0.88$, Table 3, Fig. 1), nor was glucose a significant predictor in a univariable logistic analysis (p= 0.88, Table 3). The glucose ratio was significantly lower in SI and VI patients compared to NI, and lower in VI compared to SI (.5 vs.53, $p=0.03$, Table 3, Fig. 1). It was also a significant predictor in a logistic analysis ($p=0.01$) with a pseudo-$R^2$ of 0.03 (Table 3). However, glucose ratio analyses should be interpreted cautiously due to missing data, with 50% of blood glucose values missing within 30 minutes of CSF sampling. Figure 2 illustrates that as CSF glucose levels decrease, so does the proportion of NI patients. Overall, these findings provide limited evidence that glucose can differentiate between SI and VI.

### CSF Cellularity

No significant differences in granulocyte or monocyte levels were observed between SI and VI patients. In a univariable logistic analysis, granulocytes were not a significant predictor of SI vs VI ($p=0.14$), whereas monocytes were significant ($p=0.04$), yielding a pseudo-$R^2$ of 0.02. The 1D and 2D granulocyte delta were not meaningful predictors of VI in a univariable logistic analysis with a $p=0.15$ and $p=0.17$, respectively.

The GE ratio was significantly higher in VI patients compared to SI ($p=0.03$) but was not a significant predictor of VI in a univariable logistic analysis (pseudo-$R^2 = 0.0009$, $p=0.73$). The GM ratio was higher in SI and VI patients compared to NI patients, and higher in VI compared to SI patients, but did not achieve significance as a predictor yielding a pseudo-$R^2$ of 0.009 ($p=0.08$). Visually, the proportion of SI patients seem to increase with higher GE ratios compared to VI, although any trends in the GM ratio were difficult to discern (Fig. 2). Median differences in cell counts and from cell counts derived variables between SI and VI were small; in a logistic analysis only monocytes contributed, weakly explaining variance (1.78%).

A linear regression model predicting CSF granulocyte counts, using all CSF variables as predictors, yielded a pseudo-$R^2$ of.75, indicating that 75% of the observed variance in CSF granulocyte counts can be explained by other CSF variables. Notably, the same model applied to only SI and VI patients yielded an even higher pseudo-$R^2$ of.81. This suggests considerable collinearity in EVDI surveillance CSF variables, with granulocytes and monocytes being most tightly coupled (pseudo-$R^2$ =.79).

### Blood derived and clinical parameters

C-reactive protein (CRP) levels were significantly higher in SI patients as compared to VI patients ($p=0.02$, Table 3). No other blood-derived parameters showed significant differences among the three groups, nor were any significant predictors in a univariable regression analysis. Similarly other clinical parameters including GCS and temperature did not differentiate between SI and VI patients in univariable logistic analysis. Thus, while CRP demonstrated median differences between the groups, it was noted higher noted in the SI group and likely reflects its current use in clinical decision making, however incorrectly.

### Prediction models for culture confirmed EVDI

Multivariable logistic regression was utilized to assess covariates that were found to be significant in a univariable setting (Table 3). A model including all covariates with $p < 0.2$ achieved a pseudo-$R^2$ of only 0.06, indicating a low ability to discriminate between suspected and confirmed EVDIs. Following model reduction by significance, CSF lactate and log-transformed CRP were the only retained covariates, yielding a pseudo-$R^2$ of 0.058. Interestingly, CRP exhibited an inverse relationship, being higher in patients with suspected EVDI (Table 3). Overall, only CSF lactate and CRP contributed significantly to the predictive model, but performance was low, rendering it ineffective in distinguishing between suspected and culture-confirmed EVDI.

In a tree regression analysis using the same covariates, only CSF lactate and CSF granulocytes were retained, with cutoffs of CSF lactate $>8.9$ together with CSF granulocytes $>1523$ yielding a 62% probability of VI. However, only 3 VI patients (4%) met the lactate criterion, making the model non-viable in a clinical setting. Collectively, these findings indicate that no cutoff with adequate sensitivity and specificity can be established for routinely analyzed EVDI surveillance parameters based on the extensive data from our cohort.

### Contaminations

Patients with contaminations were compared to those in the NI group, who showed no positive CSF cultures, to assess the possibility of patients classified as contaminations constituting true positives. CRP levels were higher in the contamination group and served as a statistically significant predictor of contamination in a univariable logistic regression analysis. Additionally, patients with contaminations experienced longer ICU stays and more days of catheterization. No other biochemical or clinical parameters were significantly different between contaminants and other NI patients. Given that CRP is not correlated with EVDIs in our cohort, we consider it unlikely that these findings represent patients with missed infections. Contaminations are likely linked to the duration of catheterization, and true infections are unlikely to go unnoticed with our rigorous surveillance, these findings are deemed clinically insignificant.

## Discussion

This study investigates the utility of current routine EVDI surveillance parameters and presents data on 1,828 patients over a 17-year period, encompassing 8,055 paired CSF samples and CSF bacterial cultures. To our knowledge, this is the largest study conducted to date evaluating indirect EVDI surveillance metrics in an ICU setting, and the substantial number of paired CSF samples and cultures is unique. Our main finding is that current surveillance parameters cannot distinguish true infection from aseptic CNS inflammation, thus contributing to substantial over-treatment with broad-spectrum antibiotics in the neuro-ICU.

This study categorizes participants into three defined groups, no infection (NI), suspected infection (SI), and verified infection (VI). The relative distribution of these groups is consistent with previously reported data [6, 17, 26], with our cohort demonstrating an incidence rate of 4.1% for confirmed EVDIs, which aligns with the lower end of the incidence rates reported in the literature [4, 5]. Specifically, patients receiving EVDI targeted antibiotics due to suspected infection (albeit clinically interpreted as true infections in many studies) constitute 30% of all EVD patients in our cohort. Importantly, although this group represents only 3% of the total ICU population at our institution, the EVDI targeted antibiotics administered to them accounted for 15% of the total antibiotic usage among *all* ICU patients admitted between 2006 and 2023, measured in days of treatment. Given this considerable issue, the currently most pressing clinical question and challenge that we address is how to differentiate between suspected and true infections, and is thus, the focus of this study. The validity of the three groups is essential for our analysis.

The validity of the groups requires some discussion. NI and SI groups have the risk of false negatives cultures. Given the serial nature of our surveillance with CSF samples drawn at least two times a week, we find it highly unlikely that a false negative, but clinically relevant infection, will remain so over time. Prior antibiotic therapy may increase false negative rates [27]; however, the frequency of non-EVDI targeted antibiotic use within 24 hours before CSF sampling was not significantly higher in SI patients compared to VI patients, suggesting a limited contribution of this as a cause of false negatives in our cohort. That cultures are repeatedly negative, but represent an underlying true infection with clinical implications is improbable, but not impossible, but would question CSF cultures position as the current gold standard. The VI group could represent false positives where cultures are truly contaminants, potentially confounding the comparison of SI vs VI. The few cases of cultured Micrococci and Bacillus spp., despite being clinically judged as true infections, may represent such cases as they are generally indolent. Although again possible, there is currently no way to ascertain this, as common contaminants are also known common true pathogens. This is reflected in our microbiological findings (Table 2), where the pathogenic and contamination profiles were similar for gram positive bacteria, both being dominated by coagulase-negative staphylococci which aligns with what is generally seen in the literature [20, 28].

The substantial contamination group in this study requires special consideration, as miss-classification could heavily affect the VI group. The contamination group is clinically judged as NI but where positive cultures are unexpectedly found, or in a few cases SI patients who exhibited positive cultures with clear temporal disconnect from the treatment period and assessed clinically as contaminations. Thus, we classified sampling contamination as positive cultures with no EVDI targeted antibiotic therapy given. For each individual case, it was the composite clinical judgment of the infectious diseases consultant and the intensivist adhering to local guidelines that decided what is a contamination and refrained from starting antibiotic therapy. No clinical or biochemical parameter in the contamination subgroup differed substantially with NI subgroup in large, with the marginal exception of CRP. Moreover, resampling produced negative cultures in 70 out of 75 contaminant samples, again suggesting a single sampling contamination. Five patients had repeated positive cultures which could suggest a true low virulent infection, however, given no clinical or biochemical signs and no EVDI targeted antibiotic therapy, suggest that catheter or proximal port colonization is more likely. This together highly suggests that our contamination group is in large but not conclusively correctly identified, and will not be missing true positive VIs. In aggregate, the generalization of our results will likely be related to how well our combined SI/VI group is congruent with others, more than culture based miss-classifications given that contaminants are correctly identified.

The SI and VI groups are by nature not yet differentiated at the time of antibiotic instigation, as it will take hours to days to finalize cultures [21]. This combined group is identified during the study period according to national guidelines and local practice followed in our ICU where progressive pleocytosis, increasing CSF lactate, decreasing CSF glucose, and clinical deterioration are suggestive of EVDI. Consequently, EVDI targeted antibiotic therapy was initiated in patients presenting with both clinical and biochemical signs consistent with infection, following established recommendations. Compliance to these recommendations was, for both the initiation and continuation of treatment, controlled by daily infectious disease consults. This homogeneity is reflected in Table 3 where the NI group is seen very different from the SI and VI groups. Moreover, although our study data are in part prior to 2017, our historical local practice and presented EVDI classifications are highly in line with recommendations issued by the IDSA in 2017 [6] and the national guidelines differ little from international consensus. In aggregate, we believe our groupings and findings to be clinically relevant and generalizable.

In our cohort, CSF lactate emerged as the most effective, albeit weak, parameter for differentiating between SI and VI. While prior studies have indicated elevated lactate levels in patients with EVDIs, Citerio et al. found no significant differences in lactate levels between suspected and confirmed cases [6, 17, 29, 30]. In our study, tree regression identified a lactate cut-off of $>8.9 mmol/L$ for EVDIs, which is significantly higher than the commonly cited threshold of $>4.0 mmol/L$ [6, 29]. Notably, 33% of all patients, including 25% of NI group, exceeded lactate levels of 4.0*mmol*/*L*, thus, suggesting that elevated levels may not only indicate infection but also represent part of the natural disease course in many neurocritically ill patients. Furthermore, it suggests that adherence to a threshold of 4.0*mmol*/*L* may contribute to the significant overtreatment with broad-spectrum antibiotics in this patient group. Given the significant overlap in lactate levels between SI and VI patients (Table 3), along with lactate being highly explained by other CSF variables, it is clear that current thresholds are inadequate for reliable differentiation.

The role of pleocytosis as a marker of EVDI in critically ill patients is debated, but our cohort did not show a correlation with confirmed EVDI. There were no significant differences in mean granulocyte counts or changes prior to the index date in these counts between SI and VI patients, and surprisingly, granulocytes were the least significant covariate in a full logistic model. While monocytes emerged as significant in a univariable analysis, this is likely due to their correlation with granulocytes as their role in bacterial infections is known to be limited. Additionally, 80% of the variance in granulocyte counts was explained by other CSF variables, supporting the weak association with confirmed EVDIs. Granulocyte counts were associated with the initiation of EVDI treatment, likely stemming from the historical practice at our institution of incorporating granulocyte counts into the decision-making process for treatment initiation. Other studies have identified pleocytosis as an inadequate indicator of confirmed EVDIs [26]. Our recent findings highlight potentially contributing factors to this fact, revealing significant variability in CSF cell counts when resampling after patient repositioning [8]. Moreover, pleocytosis levels in aseptic inflammation following IVH have been shown to be similar to those in EVDI patients [10, 31]. The study by Fam et al. demonstrated similar pleocytosis development in both non-infected and infected groups prior to treatment initiation. In aggregate, our current and previous findings support the notion that pleocytosis is not a reliable marker of EVDI in an ICU setting.

The pathogenesis of EVDIs is well understood, with studies emphasizing the role of hygienic measures in reducing incidence rates [24, 32]. At our institution, strict EVD protocols have been maintained throughout the study, including aseptic EVD placement in the operating theater, daily cleaning of insertion sites, and aseptic sampling from the proximal EVD port. The biochemical CSF profiles of common EVDI pathogens differ from those in community-acquired meningitis, often showing less pronounced abnormalities, where specifically coagulase-negative staphylococci may present insidiously [33–36]. We hypothesize that in a setting with rigorous hygienic measures, bacterial loads and consequently, the inflammatory responses, are reduced, making it difficult to distinguish between aseptic inflammation and true EVDIs. Our findings support this hypothesis, as our best predictive model explained only 6% of the variance in infection classification. Moreover, like the study by Chadwick et al. there were no significant differences in mortality rates between the three groups, suggesting that EVDIs may not correlate with increased mortality in settings characterized by lower bacterial loads and timely treatment [37].

The literature does not support the use of nonspecific blood markers of infection or brain injury for EVDI surveillance diagnostics, as these markers are often elevated in the ICU population [6, 9, 33]. In our cohort, we observed an inverse relationship between log-transformed CRP and VI. Higher CRP levels in the SI group likely result from clinicians interpreting elevated CRP as indicative of EVDI, although no correlation between CRP and EVDI have been established in the literature, thus enriching the SI group. Additionally, no other nonspecific blood marker was significant in differentiating between suspected and confirmed EVDI in our cohort.

Overall, this study advances the discourse on EVDI diagnostics by illustrating the limitations of existing indirect surveillance methods. It advocates for a reevaluation of these methods, supporting the need for sensitive approaches that allow for timely and direct identification of bacteria in CSF to inform clinical decision making. This shift is crucial for enhancing the accuracy of EVDI surveillance diagnostics and, most importantly, reducing overtreatment with broad-spectrum antibiotics. With this study we hope to inform future research and clinical practices in EVDI surveillance, ultimately improving patient management and outcomes in neurocritical care settings.

### Limitations

We recognize several limitations of this study. Although this study is the largest to date evaluating biochemical CSF profiles in suspected versus culture confirmed EVDI patients, it is limited to a single center and is retrospective. Moreover, there were varying degrees of missing data requiring imputation methods where missing at random cannot be ensured. Given that we have had the same electronic ICU patient data management system over the whole study period, missing data due to data retrieval problems will be limited, and missing data will represent not sampled or not given. Specifically for blood glucose, the extent of missing within 30 minutes of CSF sampling data warranted exclusion from multivariable models. A model using interpolated times series data of glucose curves to address this was evaluated (as it is sampled at least every four hours in our unit) but was likely to introduce bias. To assess changes in lactate and granulocytes leading up to the index date, exponential approximation was used for interpolating data where no samples were taken, thereby introducing an uncertainty for these variables. The routine management and care of EVDs in our ICU may differ from other institutions, potentially affecting incidence rates and the observed low virulence of our confirmed EVDI cases, and results may therefore not be fully generalized to other units. Finally, due to the ambiguity of clinical and biochemical signs associated with EVDIs, and the inherent uncertainty introduced by the possibility of false negative or positive cultures, there is a risk of infection status miss-classification in this study.

## Conclusion

In this study we evaluate the currently accepted EVDI surveillance metrics in, to our knowledge, the largest cohort of paired CSF samples and cultures to date. We find that current surveillance metrics cannot meaningfully distinguish aseptic CNS inflammation from true EVDIs in an ICU setting. This will contribute substantially to the overall use of broad-spectrum antibiotics in the ICU, and specifically to antibiotic overtreatment of EVD patients. We identify a considerable clinical problem with consequences both at the individual and population level, where more precise methods of EVDI surveillance can significantly improve antibiotic stewardship and, thus, patient care. As such, we recommend that future research focuses on evaluating new techniques, such as fast bedside sequencing methods.

## Supplementary Information

Supplementary Material 1. Central tendencies and the results from logistic and linear regression for log-transformed variables can be found in supplementary table 1.

## Data Availability

All data, including programming code for statistical analysis, is kept in safe local physical and digital storage locations in accordance with local guidelines. Data will be made available upon reasonable request to the corresponding author at marcus.badholm@ki.se.

## Abbreviations

EVD: External ventricular drain
EVDI: External ventricular drain associated infection
ICU: Intensive care unit
CNS: Central nervous system
CSF: Cerebrospinal fluid
NI: No infection
SI: Suspected infection
VI: Verified infection
IDSA: Infectious Diseases Society of America
GCS: Glascow Coma Scale
PCT: Procalcitonin
NSE: Neuron-specific enolase
GE ratio: Granulocyte to erythrocyte ratio
GM ratio: Granulocyte to monocyte ratio
SD: Standard deviation
IQR: Interquartile range
WRS: Wilcoxon rank sum
CD plot: Conditional density plot
AIC: Aikaike information criterion
SAH: Subarachnoid hemorrhage
ESBL: Extended-spectrum beta-lactamase
MRSA: Methicillin-resistant Staphylococcus aureus
AB: Antibiotics
CRP: C-reactive protein
