# Supplementary material for: Performance of routine surveillance diagnostics of external ventricular drain-associated infections in a critical care setting: a retrospective cohort study
Source: BMC Infect Dis. 2025 May 2;25:646. doi: 10.1186/s12879-025-11006-1 (PMC12046646; doi:10.1186/s12879-025-11006-1)
Supplement: Supplementary file 1 — Supplementary Material 1. Central tendencies and the results from logistic and linear regression for log-transformed variables can be found in supplementary table 1. [file 12879_2025_11006_MOESM1_ESM.pdf]

	Central Tendency				Wilcoxon Rank Sum *(p)*		Logistic Regression *(R^2)*		Linear Regression *(R^2)*	
	**NI**	**SI**	**VI**	**Missing**	**NI vs AB**	**SI vs VI**	**NI vs AB**	**SI vs VI**	**CSF Granulocytes**	**CSF Lactate**
CSF Granulocytes ($\log_{10}$)	1.48 (.91)	1.94 (1.07)	2.21 (1.01)	0	**.0000**	.0767	**.0772**	.0148	**.0901**	**.2142**
CSF Monocytes ($\log_{10}$)	1.32 (.77)	1.69 (.81)	1.86 (.79)	5	**.0000**	.1668	**.0670**	.0094	**.0653**	**.1718**
CSF Erythrocytes ($\log_{10}$)	3.96 (1.16)	3.92 (1.14)	3.80 (1.18)	0	.4710	.5340	.0009	.0021	**.0106**	**.0304**
B Leukocytes ($\log_{10}$)	1.04 (.16)	1.06 (.18)	1.08 (.22)	5	**.0042**	.8197	.0046	.0020	.0011	**.0179**
B CRP ($\log_{10}$)	1.75 (.50)	1.92 (.46)	1.74 (.62)	5	**.0000**	.0242	**.0230**	**.0238**	.0000	.0029
B PCT ($\log_{10}$)	-.70 (.55)	-.58 (.58)	-.60 (.64)	58	.0509	.6006	.0028	.0024	.0003	.0001
B NSE ($\log_{10}$)	1.18 (.23)	1.18 (.23)	1.23 (.20)	58	.7917	.2405	.0005	.0031	.0003	.0003
B s100b ($\log_{10}$)	-.93 (.45)	-1.01 (.44)	-.94 (.47)	22	**.0009**	.2631	.0038	.0030	.0027	**.0120**

Supplementary table 1: Comparisons of central tendency (CT) between groups and results of univariable logistic and linear regression analyses for log transformed variables. CT are presented as mean (SD), or as median (IQR). Missing is number of data points missing before imputations for SI and VI patients in percent. The Wilcoxon rank sum test is presented as significance levels (p). The results from logistic and linear regression are presented as bias adjusted pseduo-R^2 (Nagelkerke). Results in bold denotes a statistical significance level of $p < 0.01$. NI, SI, and VI are no infection, suspected infection, and verified infection, respectively. AB are those treated with antibiotics for EVDI (SI and VI merged). CSF granulocytes and CSF lactate columns are the results from linear regression analyses with CSF granulocytes and CSF lactate as dependent variables. CSF is cerebrospinal fluid. B indicates results related to blood analyses. CRP is C-reactive protein. PCT is procalcitonin. NSE is neuron-specific enolase.
